# The role of nucleotide composition in premature termination codon recognition

**DOI:** 10.1186/s12859-016-1384-z

**Published:** 2016-12-07

**Authors:** Fouad Zahdeh, Liran Carmel

**Affiliations:** 1 Department of Genetics, The Alexander Silberman Institute of Life Sciences, Faculty of Science, The Hebrew University of Jerusalem, Edmond J. Safra Campus, Givat Ram, Jerusalem, 91904 Israel; 2 Hereditary Research Lab, Life Sciences Department, Bethlehem University, Bethlehem, Palestine

**Keywords:** Nonsense-mediated decay (NMD), EJC-independent NMD, NMD-triggering features, Stop codon GC content, Stop codon nucleotide composition, RNA secondary structure, Exon junction complex (EJC), Transcription termination

## Abstract

**Background:**

It is not fully understood how a termination codon is recognized as premature (PTC) by the nonsense-mediated decay (NMD) machinery. This is particularly true for transcripts lacking an exon junction complex (EJC) along their 3’ untranslated region (3’UTR), and thus degrade through the EJC-independent NMD pathway.

**Results:**

Here, we analyzed data of transcript stability change following NMD repression and identified over 200 EJC-independent NMD-targets. We examined many features characterizing these transcripts, and compared them to NMD-insensitive transcripts, as well as to a group of transcripts that are destabilized following NMD repression (destabilized transcripts).

**Conclusions:**

We found that none of the known NMD-triggering features, such as the presence of upstream open reading frames, significantly characterizes EJC-independent NMD-targets. Instead, we saw that NMD-targets are strongly enriched with G nucleotides upstream of the termination codon, and even more so along their 3’UTR. We suggest that high G content around the termination codon impedes translation termination as a result of mRNA folding, thus triggering NMD. We also suggest that high G content in the 3’UTR helps to activate NMD by allowing for the accumulation of UPF1, or other NMD-promoting proteins, along the 3’UTR.

**Electronic supplementary material:**

The online version of this article (doi:10.1186/s12859-016-1384-z) contains supplementary material, which is available to authorized users.

## Background

Nonsense-mediated decay (NMD) is a major eukaryotic surveillance mechanism that targets for degradation transcripts that harbor a premature termination codon (PTC). A primary role of this mechanism is in posttranscriptional quality control, preventing the formation of truncated proteins that are potentially detrimental to the cell [1–5]. In addition, NMD is often combined with alternative splicing to form an important regulatory program of gene expression [6–9]. Altogether, NMD is central to maintaining normal cellular activity, and disruption of its proper function is estimated to be associated with about one third of inherited genetic disorders, as well as with many forms of cancer [10–12]. However, the decision-making process at the basis of NMD, determining whether a termination codon (TC) is premature or not, is still not fully understood.

It is generally believed that the context of the TC within the transcript determines whether it is normal or premature, but the nature of the contextual signals is unclear. The leading hypothesis in mammals is that a TC is recognized as premature if the pre-mRNA harbors an intron more than 50–55 bases downstream of the TC [13, 14]. The splicing reaction usually leaves traces in the form of a protein complex called the exon junction complex (EJC), which is deposited upon the mRNA ~20–24 bases upstream of the exon-exon junction [15, 16]. It is believed that the NMD-triggering feature is the presence of EJCs in the 3’ untranslated region (3’UTR) at the time of translation termination. The underlying mechanistic explanation is that the first ribosome that scans the transcript clears EJCs from the coding sequence (CDS), but is unable to clear EJCs that are downstream enough to the TC [17] (hereinafter, 3’UTR EJCs). This model explains why the presence of a translated upstream open reading frame (tuORF) is also known to trigger NMD [18–21], as the downstream CDS likely harbors EJCs which behave, in the context of the tuORF, as 3’UTR EJCs. In fact, it was suggested that many of the NMD-targets result from short tuORFs, leading to an enrichment of transcripts with short CDS among NMD-targets [22], explaining why short CDS was also reported as NMD-triggering feature in yeast [22] and Drosophila [23].

Despite overall support, this model of PTC recognition is incomplete, as in various cases it was reported to be violated: some transcripts trigger NMD despite of not harboring 3’UTR EJCs [24–27]_ENREF_20, while others are known to evade NMD despite of presenting 3’UTR EJCs [24, 28–30] or tuORFs [31]. These exceptions brought about the idea that NMD works through several different pathways, and led to a distinction between EJC-dependent NMD – which is the NMD that degrades transcripts that harbor 3’UTR EJCs – and EJC-independent NMD, or failsafe NMD – which is the NMD that works on transcripts that lack 3’UTR EJCs [32].

An alternative model (or, more precisely, a set of similar models), known as the faux-UTR model, suggests that a normal transcript is characterized by certain signals in its 3’UTR, and that disruption of these signals triggers NMD [33]_ENREF_24. Substantial evidence supports the notion that one of the central signals that marks a transcript as normal is physical proximity between the TC and the poly(A) binding protein PABPC1 [2, 34–37], and that a TC is tagged as premature by the lack of such physical proximity. Presumably, in a normal transcript the translation termination factor eRF3 binds PABPC1, whereas anything that promotes NMD, like an EJC situated downstream of the TC, competes with the PABPC1 and allows eRF3-bound up-frameshift protein 1 (UPF1) to trigger NMD. This model not only clarifies the part that 3’UTR EJCs play in NMD, but also explains why very long 3’UTRs are also known to trigger NMD [38, 39], as the TC and PABPC1 are separated by a very long physical distance. However, as for other NMD-triggering features, a long 3’UTR does not always result in NMD [36, 40].

These models assume that UPF1, on which NMD critically depends, works through specific binding with the translation termination complex [41, 42]_ENREF_37. Further evidence, however, points to the possibility that UPF1 is not only recruited by the terminating ribosome, but is associated directly with the mRNA, and is even thought to have helicase activity allowing it to slide along it [43]. It is still debated whether this association is translation-dependent [44–46] or independent [47], but all agree that UPF1 is displaced from the CDS during translation, leading to its enrichment along the 3’UTR [20, 48]. Opinions also diverge regarding the question whether NMD-targets have excess of UPF1 molecules bound to their 3’UTR. Some report that UPF1 density along the 3’UTR is higher in NMD-targets [45, 49]_ENREF_40. Others do not see significant difference in UPF1 densities between NMD-targets and NMD-insensitive transcripts, claiming that it is not the UPF1 density that matters for NMD, but rather the density of the activated (phosphorylated) UPF1 [44].

NMD may be directly linked to the efficiency of translation termination. It was proposed that unfavorable context of the TC may affect the recruitment of terminating factors, leading to changes in the kinetics of translation termination, and eventually to ribosome stalling at the TC that is thought to be the event triggering NMD [48, 50, 51]. Combined with the presence of 3’UTR-bound UPF1, this led to the suggestion that prolonged translation termination allows more UPF1 molecules to bind to the 3’UTR and become activated via regulated phosphorylation, resulting in activation of NMD [44, 48, 51]_ENREF_43.

One of the biggest challenges in understanding PTC recognition stems from the observation that neither of the proposed NMD-triggering features characterizes a large fraction of NMD-targets. Even when all features are considered together, it was estimated that they characterize roughly 30% of NMD-targets [20]. This suggests that there may be many more yet unidentified features that are important for PTC recognition. Surprisingly, the nucleotide composition around the TC has not received much attention. Hurt et al. found that UPF1 tends to bind 3’UTRs in G-rich regions, possibly because of pausing of UPF1 scanning in these regions [20]. The same authors also reported that NMD-insensitive transcripts with long 3’UTRs are enriched in poly(A) stretches, perhaps because those stretches recruit PABPC1, thus marking the TC as normal [20]. Studies on codon usage bias show that codons near the TC tend to be AT-rich [52–54], conceivably as a result of selection against the formation of RNA secondary structures near the TC that may interfere with the recruitments of release factors and lead to improper translation termination. Yet, the nucleotide composition of the sequence upstream the stop codon in the coding region was never regarded as a potential NMD triggering feature.

Here, we wished to identify additional features that are involved in the decision-making process of NMD. For this, we have used available genome-wide data on RNA stability following UPF1 knockdown [55], and identified transcripts that are targets for degradation. Specifically, we focused on transcripts that lack 3’UTR EJCs, and therefore our targets degrade through the EJC-independent NMD. For each transcript we have computed a long list of features, and tested their relevance to degradation by evaluating whether any of them shows a unique distribution within NMD-targets, when compared to non-targets. The features we have tested include all those that had been previously described as associated with NMD, as well as many new ones, mainly measuring nucleotide composition around the TC.

We found that NMD-targets are characterized by high G content around the TC and throughout the 3’UTR. However, we noticed a group of transcripts that are not NMD-targets and yet demonstrate the same, albeit weaker, pattern of enrichment. Interestingly, these transcripts are not NMD-insensitive either, but show decreased mRNA stability following UPF1 knockdown, and are thus called ‘destabilized’. These transcripts might show instability due to factors other than NMD. Surprisingly, the 3’UTR length is similar for NMD-targets and NMD-insensitive transcripts, but it is substantially shorter for the destabilized transcripts. Based on these observations we suggest that high G content around the TC and throughout the 3’UTR leads to aberrant translation termination that triggers NMD. We show that this model has a far greater discriminative power than any other EJC-independent NMD-triggering feature known today.

## Results

### Identifying NMD-targets

The ability to identify transcripts that are NMD-targets is a critical starting point in any analysis of NMD-triggering features. NMD-targets are traditionally identified as transcripts whose characteristic behavior is affected by knocking-down a core NMD component. The most common approach is to mark as NMD-targets transcripts whose expression level is increased following UPF1-knockdown. However, this approach is expected to have a high rate of false positives due to transcripts that are secondarily up-regulated. Moreover, the false negative rate may also be high due to genuine NMD-targets whose expression level is stabilized due to feedback regulation [56]. Recently, a different approach was introduced by Tani et al. [55], who used 5’-bromo-uridine immunoprecipitation chase-deep sequencing (BRIC-seq) at four time points to measure genome-wide mRNA stability. They compared control to UPF1-knockdown samples, and marked as NMD-targets transcripts whose half-life was significantly increased following UPF1-knockdown. While this approach may still suffer from secondary effects, it is nonetheless a more direct measure of RNA stability.

**Fig. 1:**
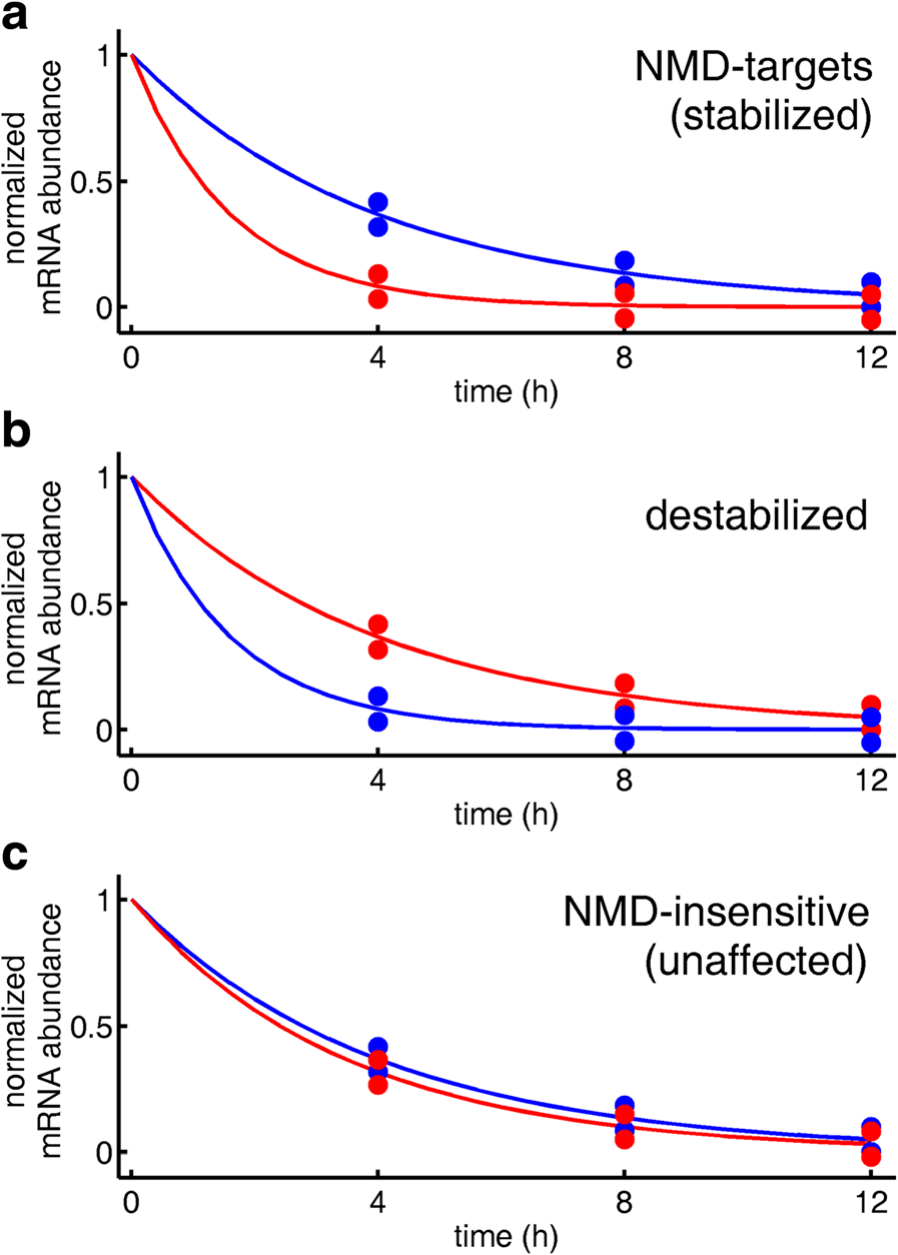
Schematic description of the transcript classification procedure. *Red* denotes mRNA abundance decay curve in the control experiments, *blue* denotes mRNA abundance decay curve following UPF1 knockdown. **a** Stabilized transcripts (NMD-targets). **b** Destabilized transcripts. **c** NMD-insensitive (unaffected) transcripts

The current analysis starts with the raw data of Tani et al. [55]. We have followed parts of their analysis pipeline, but developed a novel method to detect NMD-targets. Tani et al. estimated the decay rate of each transcript by modeling its mRNA abundance decay curve. However, decay curves of NMD-targets often deviate from a simple exponential model, reflecting a mixtures of currently degrading transcripts with older transcripts that had escaped degradation [57]. Given that Tani et al. only used four time points, their half-life estimates – especially when the decay is not a simple exponent– may have large standard errors. To circumvent this, we did not attempt to estimate decay rates, and instead developed a test that compares the shapes of the mRNA abundance decay curves and marks as NMD-targets transcripts whose decay curve following UPF1-knockdown is significantly above their control decay curve (Fig. 1a). This group of transcripts is denoted ‘stabilized’.

Theoretically, knocking down UPF1 should result in stability increase of NMD-targets, and stability stasis for NMD-insensitive transcripts. However, by using the technique above in the reverse direction we found transcripts whose stability decreases upon UPF1-knockdown, namely, their mRNA abundance decay curves following UPF1-knockdown are significantly below their control decay curves (Fig. 1b). Such transcripts are unexpected by current theory, and are denoted here ‘destabilized’. Transcripts whose decay curves did not change significantly following UPF1-knockdown were marked as ‘unaffected’, and represent transcripts that are not targeted for degradation by NMD (Fig. 1c). Finally, transcripts whose decay curves showed inconsistent behavior were left unclassified and were excluded from further analysis.

We have re-analyzed the data of Tani et al. and obtained mRNA abundance information for a total of 6080 transcripts. From these, we removed 11 whose mRNA abundance seemed to increase with time. Of the remaining 6069 transcripts, 217 (3.6%) were labeled as stabilized (NMD-targets), 169 (2.8%) were labeled as destabilized, 4329 (71.3%) were labeled as unaffected, and 1354 (22.3%) were left unclassified. In total, we could classify 4715 transcripts for which we carried out all subsequent analyses. To test the robustness of our results, we have repeated the process using a different, stricter, classification criterion totaling in 4016 classifiable transcripts (Additional file 1: Table S1).

### Generating a set of NMD-triggering features

For each of the 4715 classified transcripts we computed the following features, which were all previously suggested as characterizing NMD-targets in mammals:

- *tuorf*. A binary feature, indicating the presence/absence of translated uORFs strictly within the 5’UTR.
- *3’UTR length*, in bases.
- *3’UTR A and G content*. A and G density at the entire sequence of the 3’UTR.
- *CDS length*, in bases.
- *ALU*. A binary feature, indicating the presence/absence of ALU elements in the 3’UTR. These elements are not directly associated with NMD, but are nevertheless thought to be involved in mRNA degradation through Staufen-mediated decay [58] (see Discussion).
- *ALU density*. The density of ALU elements within the 3’UTR.

We did not consider 3’UTR EJC presence/absence as a feature, because the transcript list used by Tani et al. was based on the RefSeq database, which normally excludes transcripts that harbor 3’UTR EJCs [19]. Indeed, our data include only 14 transcripts with 3’UTR EJCs. Ten of them were classified as unaffected, whereas the other four were left unclassified. This may reflect a bias in RefSeq annotations towards the inclusion of 3’UTR EJC-harboring transcripts only if confirmed as resistant to NMD. Therefore, the transcripts we have identified in this work as NMD-target are targets of EJC-independent NMD.

These features explain just part of the differences between NMD-targets and transcripts that are not marked for degradation [20]. In order to better characterize NMD-targets, we have examined many more features, none had been previously reported to be associated with NMD. Most of these features capture various aspects of the nucleotide composition around the TC. These features are:

- *sEJC*. A binary feature, indicating the presence/absence of a ‘shallow-EJC’, meaning an EJC that comes from an intron that resides in a 3’UTR, but less than 55 bases downstream of the TC.
- *tovORF*. A binary feature, indicating whether a tuORF overlaps the main ORF.
- *Mononucleotide content*. All four nucleotide densities at the entire sequence of the 3’UTR, as well as at the last 20 bases of the CDS.
- *Dinucleotide* content. All 16 dinucleotide densities at the entire sequence of the 3’UTR, as well as at the last 20 bases of the CDS.
- *Nucleotide runs*: The number of nucleotide runs (three or more consecutive identical nucleotides) at the entire sequence of the 3’UTR, as well as at the last 20 bases of the CDS. This number was compared to the number expected by chance based on the nucleotide densities, and the resulting *χ* ^2^ -statistics were also used as features.

We ended up with a list of 63 different features. It is expected that this set of features contains many inter-dependencies, and we have therefore removed features that are highly correlated with other features. This had left us with a set of 43 non-redundant features, of which four are binaries (Additional file 1: Table S2).

### Instable transcripts are characterized by high GC content around the termination codon

**Fig. 2:**
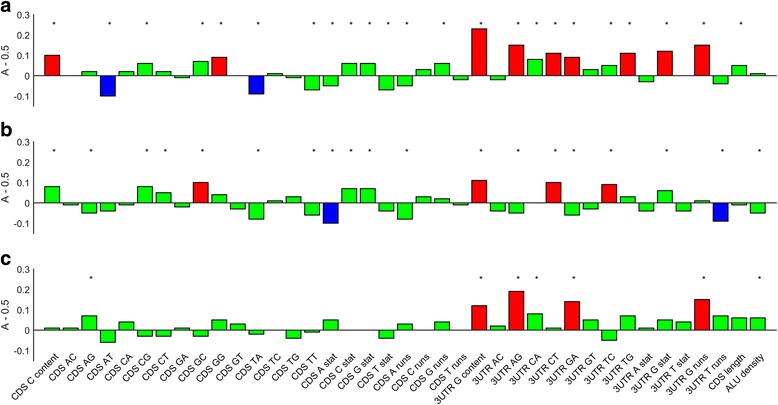
The magnitude A − 0.5, where *A* is the effect size of each non-binary feature in the non-redundant feature set. *Red* depicts features with effect size *A* − 0.5 ≥ 0.1, *blue* depicts features with effect size *A* − 0.5 ≤ − 0.1, and *green* depicts features with effect size − 0.1 < *A* − 0.5 < 0.1. **a** NMD-targets are compared to unaffected transcripts. **b** Destabilized transcripts are compared to unaffected ones. **c** NMD-targets are compared to destabilized transcripts

**Fig. 3:**
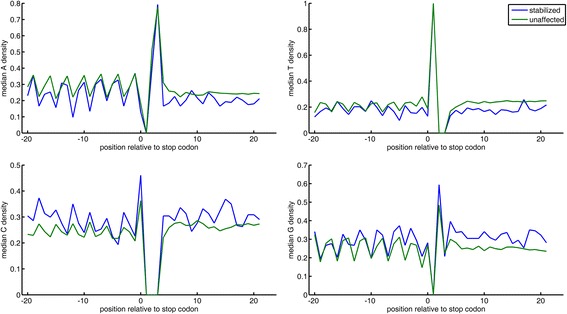
Mononucleotide densities around the TC in stabilized and unaffected transcripts

We next wished to find which features display a different distribution in stabilized transcripts (NMD-targets) when compared to unaffected ones. For the non-binary features we used the Mann–Whitney *U*-test, whereas for the binary features we used the Fisher exact test. In both cases the *p*-values were FDR-corrected. None of the binary features was found to be significantly different between the two groups of transcripts (Additional file 2: Figure S1A, Additional file 1: Table S3), but many non-binary features did. However, we noticed that the actual difference in the distributions between the two groups is typically very small. In order to account for this, we computed for each non-binary feature the non-parametric common language effect size [59]. For a feature measured for samples coming from two groups *C*_1_ and *C*_2_, the effect size *A* is defined as the probability that the feature value for a random sample from *C*_1_ would be higher than its value for a random sample from *C*_2_. Therefore, the closer *A* is to 0.5, the weaker is the effect. In total, 11 features were found to be both significantly different between stabilized and unaffected transcripts (*P* < 0.05) and to have high effect size (|*A* − 0.5| ≥ 0.1) (Fig. 2a, Additional file 1: Tables S2 and S4). Interestingly, all of them describe the nucleotide composition around the TC. At the 3’UTR side they include G content, several dinucleotide contents (AG, GA, CT, and TG), the number of G-runs, and the *χ* ^2^ G-run statistic. At the CDS side they include C content, and several dinucleotide contents (GG, AT and TA). All these features point at higher GC content upstream to a PTC as well along the entire 3’UTR (Fig. 3). Differences in 3’UTR G content between stabilized and unaffected transcripts are compatible with a recent study that found a preference for G in UPF1 binding sites at the 3’UTR of putative NMD-targets [20]. However, binding of UPF1 is unlikely to be the reason for this enrichment, as high G content extends also to the CDS, and significant enrichment is also observed for C’s.

We have repeated the above analysis to compare destabilized transcripts with unaffected ones, and found a total of six non-binary features that are significantly different and with high effect size (Additional file 1: Tables S2 and S4). Two of them (3’UTR G and CT contents) are also the features that are most discriminative between stabilized transcripts and unaffected ones (Additional file 1: Table S2). More generally, all these non-binary features show a similar trend of high GC content around the TC for stabilized and destabilized transcripts. This suggests that this characterizes transcripts that are more prone to stability modifications, regardless of the nature of the specific degradation pathway.

### NMD-targets are characterized by high G content around the termination codon

Although stabilized and destabilized transcripts show similar nucleotide composition around the TC, we noticed that some features behave differently in the two groups (Fig. 2a-b). In order to test whether any of the features discriminates directly between stabilized and destabilized transcripts, we have carried out the above analysis for a third time, now comparing destabilized transcripts with stabilized ones. We found that four such features, all related to 3’UTR G content (3’UTR G, AG, GA contents and 3’UTR G runs; Fig. 2c). This suggests that NMD-targets have higher G content around the TC, particularly along the 3’UTR. Supporting our observation that G content is elevated on both sides of the TC, we note that the dinucleotide GG is significantly enriched upstream to the TC of stabilized transcripts, but not in destabilized ones.

Among the binary features, the only one that significantly separates destabilized transcripts from unaffected ones is the presence of ALU elements in the 3’UTR (Additional file 2: Figure S1A, Additional file 1: Table S3), suggesting a strong depletion in destabilized transcripts. However, this effect is attributed to the shorter 3’UTR of destabilized transcripts, as the ALU density along their 3’UTR shows low effect size when compared to stabilized transcripts (*A* − 0.5 = 0.04) and to unaffected ones (*A* − 0.5 = 0.05).

Our results suggest that true NMD-targets are characterized by elevated G content around the TC, especially along the 3’UTR. To test the robustness of this conclusion, we have repeated the above analyses using the strict classification criterion. We observe qualitatively similar results (Additional file 2: Figures S1B, S2), although the smaller size of the stabilized and destabilized groups reduce the statistical power of the analysis. In addition, we wanted to account for the possible scenario that a transcript in our data set appears as lacking 3’UTR EJC in Refseq, and that another transcript that is indistinguishable by the BRIC-seq setup does harbor 3’UTR EJC but is absent in Refseq. To this end, we have examined all transcripts against the more permissive Ensembl database, and found that 178 out of the 217 NMD-targets (82%) do not have any isoform with 3’UTR EJCs in Ensembl. Repeating the analysis above for the reduced set of these 178 NMD-targets led to qualitatively similar results (Additional file 2: Figure S3).

### More RNA secondary structures near the TC of NMD-targets

Why high G content around the TC promotes NMD? We suggest that high G content around the TC increases the likelihood of the formation of secondary structures there, which hinders normal translation termination. Several examinations of codon usage bias towards the gene end had found a decrease in G in normally translated transcripts. Early works on *E.coli* genes revealed that they tend to end with AT-rich codons [53]. This was shortly followed with identical observations in *B. subtilis* [52] and yeast [54]. It has been previously shown that the presence of mRNA secondary structures can stall the ribosome during the elongation phase [60], or even lead to translation abortion [61]. Impeding the kinetics of translation termination may lead to a near cognate tRNA recognizing the termination codon as a sense codon and reads through it [62]. By this hypothesis, high G content near the gene end promotes the formation of RNA secondary structures, which interfere with normal translation termination and activates NMD. Enrichment in G along the 3’UTR even in regions that are far from the TC may have other reasons, which will be detailed in the Discussion.

We therefore wanted to test whether stabilized transcripts are associated with increased stability of secondary structures around the TC. For this, we used RNAfold [63] to compute the minimum free energy (MFE) around the TC. Since MFE is negative, we used its absolute value so that higher values are indicative of more stable secondary structures. We found that MFE absolute values are significantly different between stabilized, unaffected, and destabilized transcripts upstream to the TC (*P* = 3.1 ⋅ 10^− 9^, Kruskal-Wallis test) and along the 3’UTR (*P* = 3.4 ⋅ 10^− 6^, Kruskal-Wallis test). We then used Dunn’s test to find which transcript groups significantly differ from each other (Table 1), and computed their corresponding nonparametric common language effect size. We found that stabilized transcripts have significantly higher MFE absolute value than unaffected transcripts both upstream to the TC and along the 3’UTR, whereas destabilized transcripts only differ from unaffected ones upstream to the TC. This result fully agrees with our inference from nucleotide composition, and suggests that NMD-targets harbor a higher density of stable RNA secondary structures around the TC and, especially, along the 3’UTR.

**Table 1:** Comparison of MFE absolute values between stabilized, unaffected, and destabilized transcripts, both upstream to the TC and along the 3’UTR

Compared transcripts		Dunn test			Effect size
		Q value	Critical Q	Decision	
Stabilized vs. unaffected	Upstream to the TC	5.45	2.38	Reject *H*_0_	0.11
	Along the 3’UTR	4.71	2.38	Reject *H*_0_	0.09
Stabilized vs. destabilized	Upstream to the TC	1.17	2.38	Fail to reject *H*_0_	0.03
	Along the 3’UTR	4.36	2.38	Reject *H*_0_	0.14
Destabilized vs. unaffected	Upstream to the TC	3.30	2.38	Reject *H*_0_	0.07
	Along the 3’UTR	2.28	2.38	Fail to reject *H*_0_	0.05

*H*_0_ represents the null hypothesis of no difference between the two transcript groups. Effect size is defined as |*A* − 0.5|, where *A* is the common language effect size

In order to make the above analysis more quantitative, we wished to use a measure for the level of stability change of a transcript upon UPF1 knockdown, and to test whether it is correlated with the absolute value of MFE. To this end, we defined a measure for the stability change of a transcript (see Methods), and used it to divide the stabilized transcripts into three groups (small, moderate, and large stability change). We used Kruskal-Wallis test to check whether these groups are characterized by different absolute values of MFE, and found that while MFE absolute values upstream to the TC are not related to the strength of the stability change, those along the 3’UTR certainly do (*P* = 4.0 ⋅ 10^− 3^, Kruskal-Wallis test; Table 2). Moreover, transcripts with large stability change are characterized by significantly more stable RNA secondary structures along their 3’UTR than transcripts with small and moderate stability change. Although transcripts with moderate stability change show statistically indistinguishable MFE absolute values than those of small stability change, they are nevertheless slightly higher (Table 2). Similarly, while the three groups of transcripts show statistically identical MFE absolute values upstream of the TC, there is still a clear trend of higher values related to larger stability change. These results suggest that higher densities of stable RNA secondary structures around the TC and particularly along the 3’UTR are associated with higher stability change of the transcript following UPF1 knockdown.

**Table 2:** Comparison of MFE absolute values between transcripts with small, moderate, and large stability change, both upstream to the TC and along the 3’UTR

Compared transcripts		Dunn test			Effect size
		Q value	Critical Q	Decision	
Large vs. small stability change	Upstream to the TC	0.55	2.38	Fail to reject *H*_0_	0.03
	Along the 3’UTR	3.29	2.38	Reject *H*_0_	0.15
Large vs. moderate stability change	Upstream to the TC	N/A	2.38	N/A	0.02
	Along the 3’UTR	2.03	2.38	Fail to reject *H*_0_	0.10
Moderate vs. small stability change	Upstream to the TC	0.17	2.38	Fail to reject *H*_0_	0.01
	Along the 3’UTR	1.23	2.38	Fail to reject *H*_0_	0.06

*H*_0_ represents the null hypothesis of no difference between the two transcript groups. Effect size is defined as |*A* − 0.5|, where *A* is the common language effect size

### Validating the model on independent NMD-target set

To provide further support to our model, we had examined an independent set of experimentally validated EJC-independent NMD-targets. For this, we used a recent experiment by Schmidt et al. [64], who identified endogenous transcripts that are cleaved by SMG6 using Parallel Analysis of RNA Ends (PARE). These transcripts are potential NMD-targets, as cleavage by SMG6 is the initial step in NMD [65, 66]. Overall, Schmidt et al. identified 418 transcripts that are potential NMD-targets. Of these, we identified 171 transcripts that lack 3’UTR EJCs and are therefore potential EJC-independent NMD-target (see Methods). We have used this set of transcripts as our validation set, as well as a subset of 51 transcripts that are also up-regulated upon knocking down UPF1, SMG6, or both. We compared these EJC-independent NMD-targets to a list of NMD-insensitive transcripts that we prepared by taking the canonical transcripts (i.e., those with the longest CDS) of all human genes (hg19 RefSeq annotations), excluding genes that are reported as NMD-targets by Tani et al. or by Schmidt et al. (14,983 transcripts in total).

**Fig. 4:**
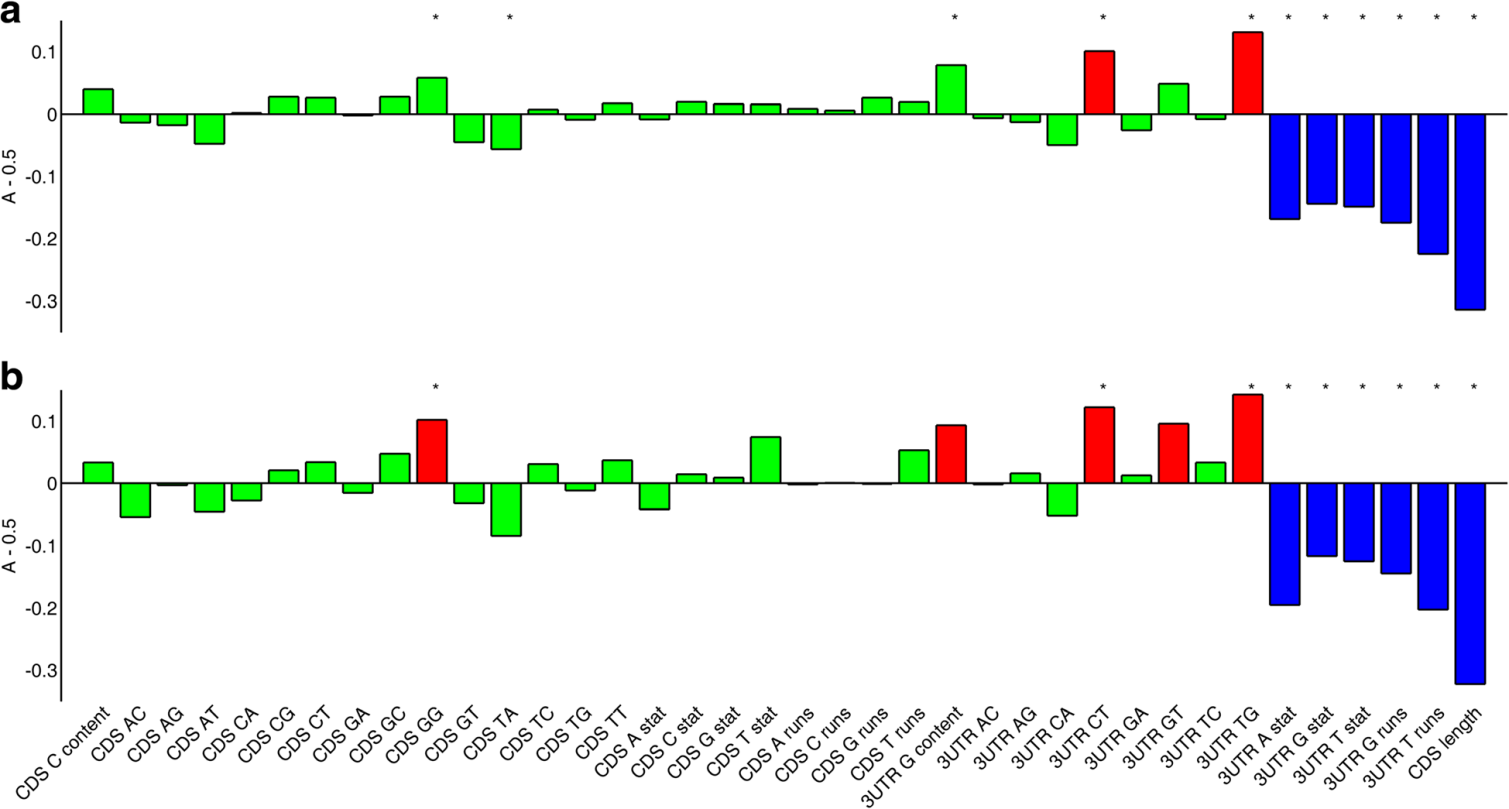
The magnitude *A* − 0.5, where *A* is the effect size of all non-binary features in the non-redundant feature set. *Red* depicts features with effect size *A* − 0.5 ≥ 0.1, *blue* depicts features with effect size *A* − 0.5 ≤ − 0.1, and *green* depicts features with effect size − 0.1 < *A* − 0.5 < 0.1. **a** Full validation set of 171 ECJ-independent NMD-targets compared to NMD-insensitive transcripts. **b** Validation set of 51 EJC-independent NMD-targets that are also up-regulated upon SMG6/UPF1 knockout compared to NMD-insensitive transcripts

Analyzing this set of EJC-independent NMD-targets, we noticed that they tend to be short, both in the CDS and in the 3’UTR (Fig. 4; Additional file 1: Table S5), possibly suggesting previously unnoticed bias in the experiment. Due to their short 3’UTR, they are depleted in nucleotide runs, reflected by low values of the *χ* ^2^-statistics. Reassuringly, this set of NMD-targets show very similar nucleotide composition to the one we observed for our set of NMD-targets, namely higher C and particularly G contents upstream to the TC and throughout the 3’UTR (Additional file 1: Table S5).

## Discussion

One of the challenging tasks in studying NMD is the genome-wide identification of its targets. Most studies equate NMD-targets with transcripts whose expression level increases following UPF1 knockdown [18–20]. However, the complex networks of protein and gene interactions lead to many indirect dependencies on UPF1 levels. As a result, transcripts that increase their expression level following UPF1 knockdown are not necessarily NMD-targets, and transcripts that are NMD-targets may seem insensitive to UPF1 knockdown [56]. In addition, expression levels show high variability among cell populations and across cell types, and the efficiency of NMD varies between cells and under different physiological conditions [67–70]. As a result, different studies identified only partially overlapping sets of NMD-targets. In order to reduce the fraction of misidentified transcripts, we have defined NMD-targets as transcripts whose mRNA half-life increases following UPF1 knockdown. While it is still plausible that some NMD-targets would be misidentified by this criterion, it is nonetheless a more direct approach, and less prone to secondary effects [71].

We have used Tani et al. data of mRNA half-life alteration following UPF1 knockdown [55], and developed our own technique to identify NMD-targets. It is important to reiterate what these targets really are. First, NMD is known to operate through EJC-dependent and EJC-independent pathways [32]. In order to be compatible with Tani et al.’s transcript nomenclature, we have used a transcript database (RefSeq) that is heavily depleted with 3’UTR EJC-bearing transcripts [19]. In total, among the 4715 transcripts that could be classified, only 14 harbor 3’UTR EJCs. Hence, the current study focuses on 3’UTR EJC-independent NMD-targets. Second, UPF1 is not critical only to NMD, but also to Staufen-mediated mRNA decay (SMD) [72]. So what we call here NMD-targets are in fact transcripts that are degraded by either SMD or 3’UTR EJC-independent NMD. We assume, however, that NMD-targets form the majority of these degraded transcripts.

In addition to stabilized (NMD-target) and unaffected (NMD-insensitive) transcripts, we have identified a third group of transcripts that are destabilized upon UPF1 knockdown. Such transcripts are instable in the absence of NMD, possibly suggesting that their stability is maintained through the suppression of certain NMD-targets. It remains to be studied what is the mechanism that regulates the stability of this unique group of transcripts.

We found that EJC-independent NMD-targets have high G content upstream to their TC and throughout their 3’UTR. The most discriminative feature of NMD-targets is 3’UTR G content (*A* − 0.5 = 0.23, *P* = 2.1 ⋅ 10^− 28^, Additional file 1: Table S4). While destabilized transcripts also display high 3’UTR G content (*A* − 0.5 = 0.11, *P* = 3.4 ⋅ 10^− 6^), it is still significantly lower than that of true NMD-targets (*A* − 0.5 = 0.12, *P* = 3.8 ⋅ 10^− 4^). This enrichment in G is also observed upstream to the TC of NMD-targets, although to a lesser extent than the enrichment along the 3’UTR. For example, the dinucleotide GG is enriched upstream of the TC in NMD-targets (*A* − 0.5 = 0.10; *P* = 7.5 ⋅ 10^− 6^), but not in destabilized transcripts (*A* − 0.5 = 0.04; *P* = 9.6 ⋅ 10^− 2^). This result was validated using an independent set of NMD-targets (Fig. 4, Additional file 1: Table S5). We showed that the enrichment of G nucleotides in NMD-targets results in higher density of stable RNA secondary structures around the TC (Table 1), and that NMD efficiency increases with the density of these secondary structures (Table 2). While the power of 3’UTR MFE to discriminate stabilized transcripts from unaffected ones (*A* −0.5 = 0.09) is lower than that of 3’UTR G content (A − 0.5 = 0.23), the density of 3’UTR MFE values (normalized to 3’UTR length) has a comparable discriminative power as the 3’UTR G content (*A* − 0.5 = 0.22, *P* = 1.1 ⋅ 10^− 27^ stabilized versus unaffected; *A* − 0.5 = 0.11, *P* = 2.5 ⋅ 10^− 04^ stabilized versus destabilized).

The effect of 3’UTR G content on NMD is probably not fully explained by the formation of secondary structures, as they may form quite far from the TC. There are other arguments suggesting that UPF1 activity is directly related to the G content along the 3’UTR. First, it was observed that UPF1 binding sites have high G content [20]. Second, UPF1 exhibits a helicase activity, and helicases are known to pauses at G-rich regions [43]. Therefore, Hurt et al. asserted that G-runs along the 3’UTR of NMD-targets block UPF1 scanning and lead to its accumulation at the 3’UTR [20]. However, this explanation may be challenged. First, it was shown that UPF1 binding is not an indicator of NMD activity, as both hyper-phosphorylated and hypo-phosphorylated UPF1 bind to the same sites, but it is only the hyper-phosphorylated UPF1 that is relevant to NMD [44]. Second, the claim that UPF1 binding sites are G-rich was not replicated in a recent study by Zund et al. [48]. We believe that an alternative model is also plausible, by which high G content along the 3’UTR increases the propensity of forming secondary structures, which promotes the recruitment of various RNA-binding proteins that may interfere in different ways with the translation termination complex. We believe that further studies should be conducted in order to determine the correct explanation.

Long 3’UTRs are believed to be enriched in NMD-targets [38, 39], presumably because they extend the physical distance between the TC and the poly(A) tail. It is therefore surprising that in our data 3’UTR length is similar between NMD-targets and NMD-insensitive transcripts (*A* − 0.5 = 0.04, *P* = 0.06). Even more striking is our analysis of Schmidt et al.’s EJC-independent NMD-targets, which showed that they have significantly shorter 3’UTRs (*A* – 0.5 = − 0.27, *P* = 9.9 ⋅ 10^− 19^). These observations suggest that EJC-independent NMD is independent of the 3’UTR length. Similarly, none of the other known NMD-triggering features (such as the presence of tuORFs) are enriched in our set of NMD-targets (Additional file 2: Figure S1). We therefore conclude that it is the nucleotide composition around the TC that mainly drives EJC-independent NMD.

While several other studies identified sets of putative NMD-targets, our focus on reliable EJC-independent NMD-targets made most of them irrelevant for the current study. For example, Andersen et al. [73] identified NMD-targets by knocking down SMG6, but all his identified NMD-targets harbor 3’UTR EJCs. Also, we avoided NMD-target sets that relied on expression data. The data produced by Schmidt et al. best suits our criteria. First, it avoids the use of expression to define NMD-targets. Instead, they were defined by examining SMG6 cleavage. Second, it is not exclusively made up of 3’UTR EJC-containing transcripts, and thus includes sufficient number of targets of the EJC-independent NMD pathway.

## Conclusions

We found that transcript features traditionally associated with NMD, such as the length of the 3’UTR and the presence of tuORF, do not characterize EJC-independent NMD targets. Rather, EJC-independent NMD is likely triggered following certain nucleotide composition features that lead to elevated levels of RNA secondary structures. The main feature is high density of G nucleotides upstream of the TC, and even more so along the 3’UTR. We propose that stable RNA secondary structures that are formed around the TC or along the 3’UTR interfere with normal translation termination leading to transcript instability and to the activation of NMD.

## Methods

### BRIC-seq analysis pipeline

BRIC-seq is a method to determine RNA stability by monitoring the decrease of 5’-bromo-uridine-labeled (BrU-labeled) RNA [55, 74]. Tani et al. used BRIC-seq to measure RNA stability in two control and two UPF1-knockdown replicates by measuring the expression level of BrU-labeled RNA at four time points: 0, 4, 8, and 12 h following the RNA pulse labeling [55]. We have downloaded their BRIC-seq raw data (accession numbers: DRA000591, DRP000622, DRS001594 − DRS001618, DRX001669 − DRX001693, and DRR002251 − DRR002275) from DDBJ (http://www.ddbj.nig.ac.jp/), and followed their computational protocol of determining the normalized FPKM in each sample, as detailed in Imamachi et al. [74]. In brief, FASTQ files were filtered for low quality reads using the FASTX-Toolkits (http://hannonlab.cshl.edu/fastx_toolkit/index.html), rRNA-derived reads were filtered out via “Bowtie --un” command [75], and the remaining reads were mapped to the UCSC hg19 reference using the default settings of TopHat [75], and directed by Refseq transcripts annotation data (gtf format). Mapped reads (i.e., BAM files) were then assembled by Cufflinks [76], and the relative expression levels (FPKM) for Refseq transcripts were calculated. As recommended for such analyses [55, 74, 77], relative expression levels were scaled to that of a stable transcript (GAPDH gene) [55, 74]. These normalization steps were carried out using a Perl script obtained from Imamachi et al. [74].

### Classifying transcripts by their response to UPF1-knockdown

By the experimental setup of Tani et al., the RNA abundance of any transcript should not increase with time. To filter out transcripts in which this is not the case, we have defined ${\tau}_{i,t}=\frac{1}{2}\left({c}_{i,t}^1+{c}_{i,t}^2 - {c}_{i,t=4}^1-{c}_{i,t=4}^2\right)$ to be the mean difference between the normalized FPKM control level of transcript *i* at time *t* ∈ {8*h*, 12*h*} and at time *t* = 4 *h*. Similarly, we have defined ${\varphi}_{i,t}=\frac{1}{2}\left({k}_{i,t}^1+{k}_{i,t}^2 - {k}_{i,t=4}^1-{k}_{i,t=4}^2\right)$ to be the mean difference between the normalized FPKM UPF1-knockdown level of transcript *i* at time *t* ∈ {8*h*, 12*h*} and at time *t* = 4 *h*. We cannot make a proper hypothesis testing using only two biological replicates, but as an approximation we can treat ${\overline{c}}_{i,t}=\frac{1}{2}\left({c}_{i,t}^1+{c}_{i,t}^2\right)$ as a mean value with standard error $\Delta {\overline{c}}_{it}\approx \frac{1}{\sqrt{2}}\left|{c}_{i,t}^1-{c}_{i,t}^2\right|$, and then by means of error propagation,

$$ \Delta {\tau}_{i,t}\approx \frac{1}{\sqrt{2}}\sqrt{{\left({c}_{i,t}^1-{c}_{i,t}^2\right)}^2+{\left({c}_{i,t=4h}^1-{c}_{i,t=4h}^2\right)}^2.} $$

Similarly, $$ \Delta {\overline{k}}_{i,t}\approx \frac{1}{\sqrt{2}}\left|{k}_{i,t}^1-{k}_{i,t}^2\right| $$ and

$$ \Delta {\varphi}_{i,t}\approx \frac{1}{\sqrt{2}}\sqrt{{\left({k}_{i,t}^1-{k}_{i,t}^2\right)}^2+{\left({k}_{i,t=4h}^1-{k}_{i,t=4h}^2\right)}^2}. $$

Using these expressions, we have filtered out transcripts in which the control mRNA abundance does not decay with time. To this end, we have used the FDR-corrected *p*-values *pτ*_*i*,*t*_ = 1 − Φ(*τ*_*i*,*t*_/Δ*τ*_*i*,*t*_), where Φ(*x*) is the cumulative standard normal distribution function, and removed all transcripts for which *pτ*_*i*,*t*_ < 0.05 for any of *t* ∈ {8*h*, 12*h*}. We have repeated this procedure for transcripts in which the UPF1-knockdown samples do not seem to decay with time, using the FDR-corrected *p*-values *pφ*_*i*,*t*_ = 1 − Φ(*φ*_*i*,*t*_/*Δφ*_*i*,*t*_) (Additional file 2: Figure S4). Overall, this process led to the removal of 11 transcripts.

Next, we wished to classify the remaining transcripts according to their response to UPF1-knockdown. To this end, we have defined ${\delta}_{i,t}=\frac{1}{2}\left({c}_{i,t}^1+{c}_{i,t}^2 - {k}_{i,t}^1-{k}_{i,t}^2\right)$ as the mean difference of the normalized FPKM level of transcript *i* between the control and the UPF1-knockdown samples at time *t*. By means of error propagation,

$$ \Delta {\delta}_{i,t} \approx \frac{1}{\sqrt{2}}\sqrt{{\left({c}_{i,t}^1-{c}_{i,t}^2\right)}^2+{\left({k}_{i,t}^1-{k}_{i,t}^2\right)}^2}. $$

We defined a transcript as stabilized at time *t* if its mRNA abundance after UPF1-knockdown was significantly higher than its abundance in the control, using the FDR-corrected *p*-value *pδ*_*i*,*t*_ = Φ(*δ*_*i*,*t*_/Δ*δ*_*i*,*t*_). Likewise, we defined a transcript as destabilized at time *t* if its mRNA abundance after UPF1-knockdown was significantly lower than its abundance in the control, using the FDR-corrected *p*-value $\tilde{p}{\delta}_{i,t}=1-\Phi \left({\delta}_{i,t}/\Delta {\delta}_{i,t}\right)$ (Additional file 2: Figure S4).

Using these definitions, we have devised two criteria to classify transcripts into stabilized, destabilized, and unaffected. The standard criterion is the one that we use throughout the paper, and it looks at the mRNA abundance at times *t* = 8 *h* and *t* = 12 *h*. Specifically, the classification scheme is (Fig. 1):

- A transcript is called stabilized (NMD-target) if *pδ*_*i*,*t*_ ≤ 0.05, and also min(*k*_*i*,*t*_^1^, *k*_*i*,*t*_^2^) > max(*c*_*i*,*t*_^1^, *c*_*i*,*t*_^2^) for *t* = 8 *h*, 12 *h* (meaning, all replicates of the UPF1-knockdown samples are above all replicates of the control samples for times 8 *h* and 12 *h*).
- A transcript is called destabilized if $\tilde{p}{\delta}_{i,t}\le 0.05$, and also min(*c*_*i*,*t*_^1^, *c*_*i*,*t*_^2^) > max(*k*_*i*,*t*_^1^, *k*_*i*,*t*_^2^) for t = 8 *h*, 12 *h*.
- A transcript is called unaffected if both *pδ*_*i*,*t*_ > 0.05 and $\tilde{p}{\delta}_{i,t}>0.05$ for *t* = 8 *h*, 12 *h*.

Notably, some transcripts do not fall into any of these classes, and are consequently marked as unclassified (Additional file 1: Table S1). To test the robustness of our classification scheme, we employed a second, stricter, criterion that looks at the mRNA abundance also at *t* = 4 *h*. Specifically:

- A transcript is called stabilized (NMD-target) if *pδ*_*i*,*t*_ ≤ 0.05, and also min(*k*_*i*,*t*_^1^, *k*_*i*,*t*_^2^) > max(*c*_*i*,*t*_^1^, *c*_*i*,*t*_^2^) for *t* = 4 *h*, 8 *h*, 12 *h*.
- A transcript is called destabilized if $\tilde{p}{\delta}_{i,t}\le 0.05$, and also min(*c*_*i*,*t*_^1^, *c*_*i*,*t*_^2^) > max(*k*_*i*,*t*_^1^, *k*_*i*,*t*_^2^) for *t* = 4 *h*, 8 *h*, 12 *h.*
- A transcript is called unaffected if both *pδ*_*i*,*t*_ > 0.05 and $\tilde{p}{\delta}_{i,t}>0.05$ for *t* = 4 *h*, 8 *h*, 12 *h*.

This stricter classification scheme resulted in more unclassified transcripts (Additional file 1: Table S1).

### Measuring stability change

The stability change of transcript *i*, *S*_*i*_, is defined as the median of *δ*_i,t_ at times *t* = 4 *h*, 8 *h*, 12 *h*. We split stabilized transcripts to three groups based on their stability change value: small activity change (lower than or equal to the 33^rd^ percentile), moderate activity change (from 33^rd^ to 66^th^ percentiles), and large activity change (higher than or equal to the 66^th^ percentile).

### Generating a set of non-redundant features

In order to remove highly correlated features, we first ranked all the non-binary features based on the non-parametric common language effect size (A), measuring for each feature how strongly it differs between stabilized and the unaffected transcripts. Then, from each pair of highly-correlated features (*abs*(*R*) ≥ 0.7, where *R* is the Spearman correlation coefficient), we recursively removed the lower ranked one. The four binary features were automatically included in the non-redundant set. At the end of the process we were left with 43 non-redundant features (Additional file 1: Table S2).

When looking at tuORFs, we have made a distinction between transcripts in which the tuORF is strictly within the 5’UTR, and those in which the tuORF overlaps the main ORF. The former may repress decay due to translation re-initiation at the main ORF, while the latter wouldn’t allow this.

### Non-parametric common language effect size

For a feature *x* measured for samples coming from two classes *C*_1_ and *C*_2_, the effect size *A* is defined as the probability that the feature value of a random sample from *C*_1_ is higher than its value for a random sample from *C*_2_ [59]. Let *n*_1_ and *n*_2_ be the number of samples in classes *C*_1_ and *C*_2_, respectively. Let us look at all pairs of samples such that one is from *C*_1_ and the other is from *C*_2_. From among these pairs, let ${n}_{C_1>{C}_2}$ be the number of pairs in which *x*(*C*_1_) > *x*(*C*_2_). Similarly, let ${n}_{C_1={C}_2}$ be the number of pairs in which *x*(*C*_1_) = *x*(*C*_2_). Then, the effect size *A* is calculated by

$$ A=\frac{n_{C_1>{C}_2}+\frac{1}{2}{n}_{C_1={C}_2}}{n_1{n}_2}. $$

### Runs of nucleotides and the *χ*^2^-statistic

For every sequence *i* and nucleotide of type *s*, the probability *p* of having a run of exactly *m* consecutive appearances of *s* is

$$ {p}_{m,s,i} = {\delta_{s,i}}^m\cdot {\left(1-{\delta}_{s,i}\right)}^2, $$

where *δ*_*s*,*i*_ is the density of nucleotide *s* in sequence *i*. If the length of sequence *i*, *l*_*i*_, is sufficiently long, the expected number of runs is approximately

*E*_*m*,*s*,*i*_ = *p*_*m*,*s*,*i*_ ⋅ *l*_*i*_.

Let *O*_*m*,*s*,*i*_ be the observed number of runs of *m* consecutive appearances of nucleotide *s* in sequence *i*. In practice, we shall be interested in runs of minimum length *m* = 3, and up to the practical limit of length *m* = 7. Based on the goodness of fit test, the *χ* ^2^-statistic for sequence *i* and for nucleotide *s* is given by

$$ {\chi}_{s,i}^2={\displaystyle \sum_{m=3}^7}\frac{{\left(\sqrt{\frac{n_O}{n_E}\cdot {E}_{m,s,i}}-\sqrt{\frac{n_E}{n_O}\cdot {O}_{m,s,i}}\right)}^2}{E_{m,s,i}+{O}_{m,s,i}}. $$

Here, *n*_*O*_ = ∑_*m* = 3_^7^ *O*_*m*,*s*,*i*_ is the total number of observed *s*-runs, and *n*_*E*_ = ∑_*m* = 3_^7^ *E*_*m*,*s*,*i*_ is the total number of expected *s*-runs.

### Ribosome footprint data

Data on tuORFs were taken from https://www.ncbi.nlm.nih.gov/pmc/articles/PMC3483550.

### Predicting RNA secondary structures

We used the default parameters of RNAfold 2.1.8 [63] from the Vienna Package to predict local stable secondary structures. The minimum free energy (MFE) was computed and its absolute value was used throughout the study.

### Construction of the validation set

We used BioMart to convert Schmidt et al.’s transcript names [64] to Ensembl IDs, ending with a successful conversion of 327 out of the original 418 NMD-targets. We removed transcripts with more than one corresponding Ensembl ID, as well as transcripts that lack 3’UTR or CDS annotations.

## Additional files

Additional file 1: Table S1. Summary of transcript classification based on how their mRNA abundance decay curve is affected by UPF1-knockdown. **Table S2.** Putative NMD-triggering features examined in the current study. a Features marked by ‘Yes’ belong to the non-redundant set, meaning that they are sufficiently uncorrelated to each other. b Features marked by ‘Yes’ are significantly different between NMD-targets and unaffected transcripts ($P$<0.05, FDR-corrected *U*-test) and also have high effect size (|$A$−0.5| ≥ 0.1). c Features marked by ‘Yes’ are significantly different between destabilized and unaffected transcripts ($P$<0.05, FDR-corrected *U*-test) and also have high effect size (|$A$−0.5| ≥ 0.1). **Table S3.** The level of enrichment of each binary feature (its fraction in one class divided by its fraction in the second class). Asterisks denote significant enrichments ($P$<0.05, Fisher exact test). **Table S4.** Effect size (ES, $A$–0.5) and *P*-value (FDR-corrected, *U*-test) of the non-redundant features, for all pairwise comparisons of transcript groups. **Table S5.** Effect size (ES, $A$– 0.5) and *p*-value (FDR-corrected, *U*-test) of the non-redundant features for the two validation sets. (PDF 242 kb)
Additional file 2: Figure S1. For each binary feature (x-axis) and transcript group (colors), the bars show the percentage of transcripts in which the feature is present. Error bars indicate one binomial standard deviation. (A) The transcripts were classified by the standard classification scheme. (B) The transcripts were classified by the strict classification scheme. **Figure S2.** The magnitude *A* − 0.5, where *A* is the effect size of each non-binary feature in the non-redundant feature set, and where the transcripts were classified using the strict criterion. Red depicts features with effect size *A* − 0.5 ≥ 0.1, blue depicts features with effect size *A* − 0.5 ≤ − 0.1, and green depicts features with effect size − 0.1 < *A* − 0.5 < 0.1. (A) NMD-targets are compared to unaffected transcripts. (B) Destabilized transcripts are compared to unaffected ones. (C) NMD-targets are compared to destabilized transcripts. **Figure S3.** NMD-targets are compared to unaffected transcripts, when only transcripts that do not have isoforms harboring 3’UTR EJC were taken into account. The bars show The magnitude *A* − 0.5, where *A* is the effect size of each non-binary feature in the non-redundant feature set. Red depicts features with effect size *A* − 0.5 ≥ 0.1, blue depicts features with effect size *A* − 0.5 ≤ − 0.1, and green depicts features with effect size − 0.1 < *A* − 0.5 < 0.1. **Figure S4.** A schematic description of the different parameters used to classify transcripts into stabilized, unaffected, and destabilized. (PDF 231 kb)

## Abbreviations

CDS: Coding sequence
EJC: Exon-junction complex
MFE: Minimum free energy
NMD: Nonsense-mediated decay
PTC: Premature termination codon
TC: Termination codon
tuORF: Translated upstream open reading frame
uORF: Upstream open reading frame
