# Supplementary material for: The role of nucleotide composition in premature termination codon recognition
Source: BMC Bioinformatics. 2016 Dec 7;17:519. doi: 10.1186/s12859-016-1384-z (PMC5142417; doi:10.1186/s12859-016-1384-z)
Supplement: Additional file 1: Table S1. — Summary of transcript classification based on how their mRNA abundance decay curve is affected by UPF1-knockdown. Table S2. Putative NMD-triggering features examined in the current study. a Features marked by ‘Yes’ belong to the non-redundant set, meaning that they are sufficiently uncorrelated to each other. b Features marked by ‘Yes’ are significantly different between NMD-targets and unaffected transcripts ($P$P<0.05, FDR-corrected U-test) and also have high effect size (|$A$A−0.5| ≥ 0.1). c Features marked by ‘Yes’ are significantly different between destabilized and unaffected transcripts ($P$P<0.05, FDR-corrected U-test) and also have high effect size (|$A$A−0.5| ≥ 0.1). Table S3. The level of enrichment of each binary feature (its fraction in one class divided by its fraction in the second class). Asterisks denote significant enrichments ( [file 12859_2016_1384_MOESM1_ESM.pdf]

Supplementary Tables for the paper:

The role of nucleotide composition in premature termination codon recognition

Fouad Zahdeh, Liran Carmel

	classification scheme			
	Standard		Strict	
	Number	Percentage	Number	Percentage
Stabilized (NMD-targets)	217	3.6%	90	1.5%
Unaffected	4,329	71.3%	3,864	63.7%
Destabilized	169	2.8%	62	1.0%
Total classified	4,715	77.7%	4,016	66.2%
Total unclassified	1,354	22.3%	2,053	33.8%
**Total**	6,069		6,069	

Table S1. Summary of transcript classification based on how their mRNA abundance decay curve is affected by UPF1-knockdown.

Feature name	Present in the non-redundant set?[a]	Characterize stabilized?[b]	Characterize destabilized?[c]
sEJC	Yes	No	No
tuORF	Yes	No	No
tovORF	Yes	No	No
ALU	Yes	No	Yes
ALU density	Yes	No	No
3'UTR A content	No	No	No
3'UTR C content	No	No	No
3'UTR G content	Yes	Yes	Yes
3'UTR T content	No	No	No
CDS A content	No	No	No
CDS C content	Yes	Yes	No
CDS G content	No	No	No
CDS T content	No	No	No
3'UTR AA content	No	No	No
3'UTR AC content	Yes	No	No
3'UTR AG content	Yes	Yes	No
3'UTR AT content	No	No	No
3'UTR CA content	Yes	No	No
3'UTR CC content	No	No	No
3'UTR CG content	No	No	No
3'UTR CT content	Yes	Yes	Yes
3'UTR GA content	Yes	Yes	No
3'UTR GC content	No	No	No
3'UTR GG content	No	No	No
3'UTR GT content	Yes	No	No
3'UTR TA content	No	No	No
3'UTR TC content	Yes	No	Yes
3'UTR TG content	Yes	Yes	No

**3'UTR TT content**	No	No	No
**CDS AA content**	No	No	No
**CDS AC content**	Yes	No	No
**CDS AG content**	Yes	No	No
**CDS AT content**	Yes	Yes	No
**CDS CA content**	Yes	No	No
**CDS CC content**	No	No	No
**CDS CG content**	Yes	No	No
**CDS CT content**	Yes	No	No
**CDS GA content**	Yes	No	No
**CDS GC content**	Yes	No	Yes
**CDS GG content**	Yes	Yes	No
**CDS GT content**	Yes	No	No
**CDS TA content**	Yes	Yes	No
**CDS TC content**	Yes	No	No
**CDS TG content**	Yes	No	No
**CDS TT content**	Yes	No	No
**# 3'UTR A runs**	No	No	No
**# 3'UTR C runs**	No	No	No
**# 3'UTR G runs**	Yes	Yes	No
**# 3'UTR T runs**	Yes	No	Yes
**# CDS A runs**	Yes	No	No
**# CDS C runs**	Yes	No	No
**# CDS G runs**	Yes	No	No
**# CDS T runs**	Yes	No	No
χ^2 **3'UTR A runs**	Yes	No	No
χ^2 **3'UTR C runs**	No	No	No
χ^2 **3'UTR G runs**	Yes	Yes	No
χ^2 **3'UTR T runs**	Yes	No	No
χ^2 **CDS A runs**	Yes	No	Yes
χ^2 **CDS C runs**	Yes	No	No
χ^2 **CDS G runs**	Yes	No	No
χ^2 **CDS T runs**	Yes	No	No
**3'UTR length**	No	No	No
**CDS length**	Yes	No	No

Table S2. Putative NMD-triggering features examined in the current study. [a] Features marked by 'Yes' belong to the non-redundant set, meaning that they are sufficiently uncorrelated to each other. [b] Features marked by 'Yes' are significantly different between NMD-targets and unaffected transcripts ($P < 0.05$, FDR-corrected U-test) and also have high effect size ($|A - 0.5| \geq 0.1$). [c] Features marked by 'Yes' are significantly different between destabilized and unaffected transcripts ($P < 0.05$, FDR-corrected U-test) and also have high effect size ($|A - 0.5| \geq 0.1$).

	Stabilized vs. unaffected		Destabilized vs. unaffected		Stabilized vs. destabilized	
**Feature**	**Enrichment**	**P-value**	**Enrichment**	**P-value**	**Enrichment**	**P-value**
**sEJC**	0.7	0.7	1.2	0.8	0.6	0.4
**tuORF**	1.4	0.1	1.4	0.1	1.0	1.0
**tovORF**	0.6	0.2	1.1	0.8	0.6	0.3
**ALU**	1.0	0.9	0.5	0.002*	2.1	0.02*

Table S3. The level of enrichment of each binary feature (its fraction in one class divided by its fraction in the second class). Asterisks denote significant enrichments ($P < 0.05$, Fisher exact test).

Feature name	stabilized vs. unaffected		destabilized vs. unaffected		stabilized vs. destabilized	
	ES	P-value	ES	P-value	ES	P-value
**CDS A content**	-0.09	2.27e-05	-0.10	5.75e-05	0.01	8.27e-01
**CDS C content**	0.10	1.77e-06	0.08	6.66e-04	0.01	8.27e-01
**CDS G content**	0.08	4.82e-05	0.07	6.75e-03	0.02	5.69e-01
**CDS T content**	-0.09	1.41e-05	-0.04	8.27e-02	-0.05	1.99e-01
**CDS AA content**	-0.07	4.01e-04	-0.06	8.03e-03	-0.01	8.37e-01
**CDS AC content**	-0.00	9.88e-01	-0.01	7.54e-01	0.01	8.96e-01
**CDS AG content**	0.02	2.40e-01	-0.05	3.95e-02	0.07	4.40e-02
**CDS AT content**	-0.10	8.06e-07	-0.04	1.08e-01	-0.06	1.10e-01
**CDS CA content**	0.02	2.50e-01	-0.01	6.13e-01	0.04	3.53e-01
**CDS CC content**	0.08	3.05e-05	0.07	2.89e-03	0.01	8.27e-01
**CDS CG content**	0.06	1.21e-03	0.08	1.17e-04	-0.03	4.98e-01
**CDS CT content**	0.02	3.92e-01	0.05	3.95e-02	-0.03	3.77e-01
**CDS GA content**	-0.01	5.07e-01	-0.02	3.00e-01	0.01	8.37e-01
**CDS GC content**	0.07	3.19e-04	0.10	1.40e-05	-0.03	4.55e-01
**CDS GG content**	0.10	7.46e-06	0.04	9.64e-02	0.05	1.82e-01
**CDS GT content**	0.00	9.07e-01	-0.03	2.06e-01	0.03	3.77e-01
**CDS TA content**	-0.10	3.31e-07	-0.08	4.81e-04	-0.02	5.57e-01
**CDS TC content**	0.01	7.98e-01	0.01	7.62e-01	-0.00	9.75e-01
**CDS TG content**	-0.01	7.41e-01	0.03	1.73e-01	-0.04	2.90e-01
**CDS TT content**	-0.07	4.33e-04	-0.06	1.14e-02	-0.01	8.11e-01
χ^2**CDS A runs**	-0.05	2.77e-02	-0.10	2.86e-05	0.05	1.82e-01
χ^2**CDS C runs**	0.06	2.39e-03	0.07	5.74e-03	-0.00	9.75e-01
χ^2**CDS G runs**	0.06	2.34e-03	0.07	5.18e-03	0.00	9.95e-01
χ^2**CDS T runs**	-0.07	4.27e-04	-0.04	1.06e-01	-0.04	3.77e-01
**# CDS A runs**	-0.05	1.46e-03	-0.08	2.55e-05	0.03	2.70e-01
**# CDS C runs**	0.03	7.59e-02	0.03	1.54e-01	0.00	9.75e-01
**# CDS G runs**	0.06	2.87e-05	0.02	1.63e-01	0.04	2.40e-01
**# CDS T runs**	-0.02	2.28e-01	-0.01	3.50e-01	-0.00	9.75e-01
**3'UTR A content**	-0.20	2.38e-21	-0.17	1.68e-12	-0.01	8.37e-01
**3'UTR C content**	0.17	1.79e-17	0.15	1.52e-09	0.01	8.37e-01
**3'UTR G content**	0.23	2.12e-28	0.11	3.35e-06	0.12	3.78e-04
**3'UTR T content**	-0.19	1.50e-20	-0.10	8.26e-05	-0.10	6.10e-03
**3'UTR AA content**	-0.20	4.02e-23	-0.14	7.59e-09	-0.07	8.69e-02
**3'UTR AC content**	-0.02	4.23e-01	-0.04	1.25e-01	0.02	6.35e-01
**3'UTR AG content**	0.15	9.43e-14	-0.05	3.22e-02	0.19	7.94e-09
**3'UTR AT content**	-0.20	4.02e-23	-0.15	1.23e-09	-0.05	2.61e-01
**3'UTR CA content**	0.08	1.01e-04	0.00	9.69e-01	0.08	2.78e-02

**3’UTR CC content**	0.17	1.63e-17	0.15	5.64e-10	-0.00	9.75e-01
**3’UTR CG content**	0.18	1.25e-18	0.12	6.07e-07	0.04	2.63e-01
**3’UTR CT content**	0.11	2.71e-07	0.10	4.02e-05	0.01	9.61e-01
**3’UTR GA content**	0.09	7.50e-06	-0.06	1.38e-02	0.14	2.24e-05
**3’UTR GC content**	0.19	8.36e-20	0.12	6.00e-07	0.07	9.16e-02
**3’UTR GG content**	0.21	4.11e-25	0.13	2.32e-07	0.08	2.78e-02
**3’UTR GT content**	0.03	1.97e-01	-0.03	2.74e-01	0.05	1.82e-01
**3’UTR TA content**	-0.21	4.45e-24	-0.15	7.82e-10	-0.06	1.23e-01
**3’UTR TC content**	0.05	1.52e-02	0.09	2.29e-04	-0.05	1.99e-01
**3’UTR TG content**	0.11	4.01e-07	0.03	2.19e-01	0.07	6.33e-02
**3’UTR TT content**	-0.18	1.58e-18	-0.07	2.44e-03	-0.12	7.90e-04
χ^2**3'UTR A runs**	-0.03	2.28e-01	-0.04	1.29e-01	0.01	8.37e-01
χ^2**3'UTR C runs**	0.13	9.31e-10	0.04	8.99e-02	0.08	4.07e-02
χ^2**3'UTR G runs**	0.12	1.79e-08	0.06	9.65e-03	0.05	1.79e-01
χ^2**3'UTR T runs**	0.00	9.99e-01	-0.04	1.17e-01	0.04	3.53e-01
**# 3'UTR A runs**	-0.06	4.05e-03	-0.12	3.06e-07	0.07	5.39e-02
**# 3'UTR C runs**	0.14	2.98e-12	0.03	2.11e-01	0.11	9.57e-04
**# 3'UTR G runs**	0.15	6.43e-14	0.01	7.86e-01	0.15	8.62e-06
**# 3'UTR T runs**	-0.04	7.59e-02	-0.10	9.07e-05	0.07	8.69e-02
**CDS length**	0.05	1.18e-02	-0.01	7.86e-01	0.06	1.22e-01
**3’UTR length**	0.04	7.74e-02	-0.08	1.10e-03	0.13	2.89e-04
**ALU density**	0.01	7.28e-01	-0.05	2.44e-03	0.06	1.25e-02

Table S4. Effect size (ES, $A - 0.5$) and P-value (FDR-corrected, U-test) of the non-redundant features, for all pairwise comparisons of transcript groups.

Feature name	EJC-independent SMG6 target		Up-regulated EJC-independent SMG6 target	
	ES	P-value	ES	P-value
**CDS A content**	-0.04	1.22e-01	-0.07	1.98e-01
**CDS C content**	0.04	1.31e-01	0.03	5.75e-01
**CDS G content**	0.02	3.76e-01	0.04	4.55e-01
**CDS T content**	-0.01	6.46e-01	0.02	7.62e-01
**CDS AA content**	-0.00	8.80e-01	-0.03	5.99e-01
**CDS AC content**	-0.01	6.46e-01	-0.05	3.42e-01
**CDS AG content**	-0.02	5.58e-01	-0.00	9.85e-01
**CDS AT content**	-0.05	5.39e-02	-0.05	4.22e-01
**CDS CA content**	0.00	9.27e-01	-0.03	6.25e-01
**CDS CC content**	0.04	1.20e-01	0.05	3.69e-01
**CDS CG content**	0.03	2.53e-01	0.02	7.02e-01
**CDS CT content**	0.03	3.42e-01	0.03	5.75e-01
**CDS GA content**	-0.00	9.27e-01	-0.02	8.27e-01
**CDS GC content**	0.03	3.12e-01	0.05	4.05e-01
**CDS GG content**	0.06	1.74e-02	0.10	4.11e-02
**CDS GT content**	-0.05	5.92e-02	-0.03	5.75e-01
**CDS TA content**	-0.06	1.30e-02	-0.08	7.13e-02
**CDS TC content**	0.01	7.86e-01	0.03	5.84e-01
**CDS TG content**	-0.01	7.84e-01	-0.01	8.57e-01
**CDS TT content**	0.02	5.58e-01	0.04	5.12e-01
χ^2 **CDS A**	-0.01	7.86e-01	-0.04	4.89e-01
χ^2 **CDS C**	0.02	5.42e-01	0.01	8.43e-01
χ^2 **CDS G**	0.02	6.24e-01	0.01	9.02e-01
χ^2 **CDS T**	0.02	6.24e-01	0.07	1.65e-01
**# CDS A runs**	0.01	7.41e-01	-0.00	9.85e-01
**# CDS C runs**	0.01	7.86e-01	0.00	9.87e-01
**# CDS G runs**	0.03	1.41e-01	-0.00	9.85e-01
**# CDS T runs**	0.02	3.07e-01	0.05	1.27e-01
**3’UTR A content**	-0.12	6.06e-07	-0.12	1.17e-02
**3’UTR C content**	0.07	4.19e-03	0.08	1.31e-01
**3’UTR G content**	0.08	1.39e-03	0.09	7.13e-02
**3’UTR T content**	-0.04	1.24e-01	-0.04	4.61e-01
**3’UTR AA content**	-0.12	6.17e-07	-0.14	3.47e-03
**3’UTR AC content**	-0.01	8.19e-01	-0.00	9.85e-01
**3’UTR AG content**	-0.01	6.85e-01	0.02	8.27e-01
**3’UTR AT content**	-0.10	7.02e-05	-0.10	4.92e-02
**3’UTR CA content**	-0.05	5.39e-02	-0.05	3.69e-01
**3’UTR CC content**	0.06	2.99e-02	0.05	3.69e-01
**3’UTR CG content**	0.05	5.39e-02	0.09	7.53e-02

**3’UTR CT content**	0.10	2.31e-05	0.12	1.23e-02
**3’UTR GA content**	-0.03	3.61e-01	0.01	8.56e-01
**3’UTR GC content**	0.08	6.80e-04	0.08	1.20e-01
**3’UTR GG content**	0.06	3.02e-02	0.06	2.62e-01
**3’UTR GT content**	0.05	5.92e-02	0.10	6.50e-02
**3’UTR TA content**	-0.08	5.01e-04	-0.09	8.89e-02
**3’UTR TC content**	-0.01	7.86e-01	0.03	5.75e-01
**3’UTR TG content**	0.13	1.91e-08	0.14	3.04e-03
**3’UTR TT content**	-0.06	1.72e-02	-0.07	1.71e-01
χ^2 **3’UTR A**	-0.17	2.44e-13	-0.20	1.64e-05
χ^2 **3’UTR C**	-0.11	3.49e-06	-0.08	1.11e-01
χ^2 **3’UTR G**	-0.14	5.35e-10	-0.12	1.76e-02
χ^2 **3’UTR T**	-0.15	1.52e-10	-0.12	1.17e-02
**# 3’UTR A runs**	-0.26	2.20e-29	-0.25	2.45e-08
**# 3’UTR C runs**	-0.18	1.15e-15	-0.16	5.22e-04
**# 3’UTR G runs**	-0.17	3.20e-14	-0.14	2.89e-03
**# 3’UTR T runs**	-0.22	6.08e-23	-0.20	8.07e-06
**CDS length**	-0.31	7.84e-40	-0.32	2.30e-12
**3’UTR length**	-0.24	1.23e-25	-0.21	4.33e-06

Table S5. Effect size (ES, $A - 0.5$) and p-value (FDR-corrected, U-test) of the non-redundant features for the two validation sets.
