# Supplementary material for: The role of nucleotide composition in premature termination codon recognition
Source: BMC Bioinformatics. 2016 Dec 7;17:519. doi: 10.1186/s12859-016-1384-z (PMC5142417; doi:10.1186/s12859-016-1384-z)
Supplement: Additional file 2: Figure S1. — For each binary feature (x-axis) and transcript group (colors), the bars show the percentage of transcripts in which the feature is present. Error bars indicate one binomial standard deviation. (A) The transcripts were classified by the standard classification scheme. (B) The transcripts were classified by the strict classification scheme. Figure S2. The magnitude A − 0.5, where A is the effect size of each non-binary feature in the non-redundant feature set, and where the transcripts were classified using the strict criterion. Red depicts features with effect size A − 0.5 ≥ 0.1, blue depicts features with effect size A − 0.5 ≤ − 0.1, and green depicts features with effect size − 0.1 < A − 0.5 < 0.1. (A) NMD-targets are compared to unaffected transcripts. (B) Destabilized transcripts are compared to unaffected ones. (C) NMD-targets are compared to destabilized transcripts. Figure S3. NMD-targets are compared to unaffected transcripts, when only transcripts that do not have isoforms harboring 3’UTR EJC were taken into account. The bars show The magnitude A − 0.5, where A is the effect size of each non-binary feature in the non-redundant feature set. Red depicts features with effect size A − 0.5 ≥ 0.1, blue depicts features with effect size A − 0.5 ≤ − 0.1, and green depicts features with effect size − 0.1 < A − 0.5 < 0.1. Figure S4. A schematic description of the different parameters used to classify transcripts into stabilized, unaffected, and destabilized. (PDF 231 kb) [file 12859_2016_1384_MOESM2_ESM.pdf]

Supplementary Figures for the paper:

The role of nucleotide composition in premature termination codon recognition

Fouad Zahdeh, Liran Carmel

Figure S1

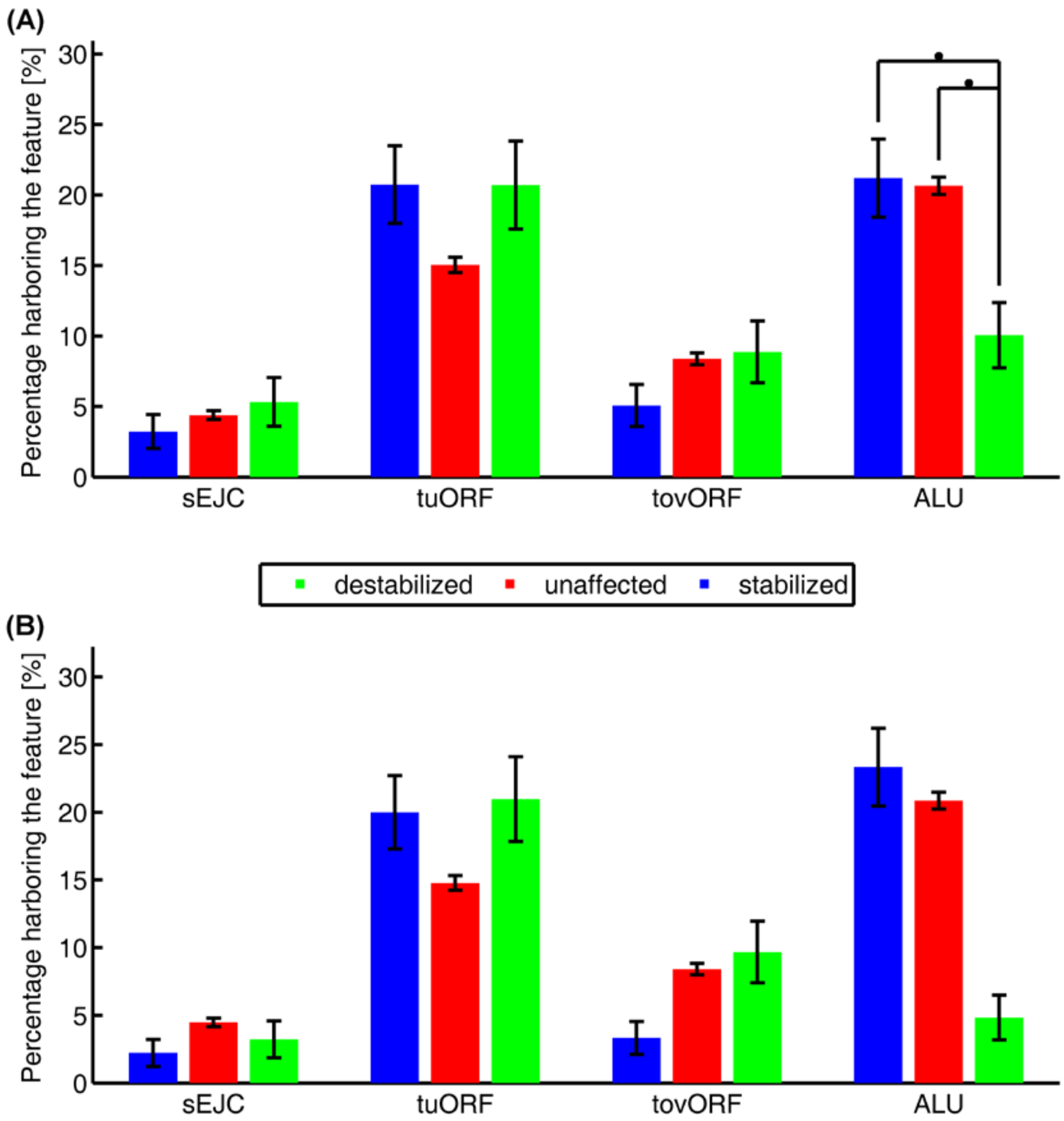
(A)
Percentage harboring the feature [%]
0
5
10
15
20
25
30
sEJC
tuORF
tovORF
ALU
destabilized
unaffected
stabilized
(B)
Percentage harboring the feature [%]
0
5
10
15
20
25
30
sEJC
tuORF
tovORF
ALU

Figure S2

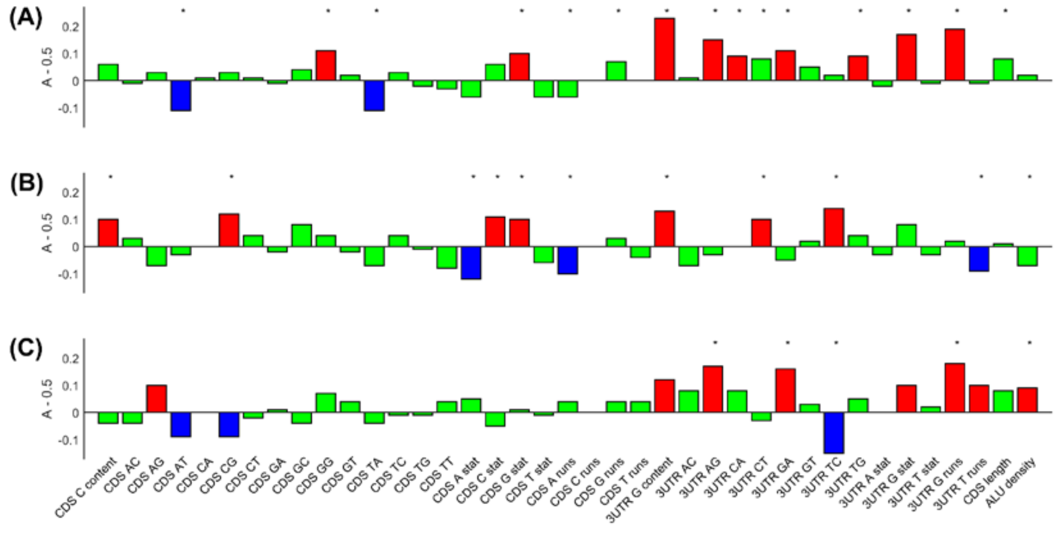

(A)
(B)
(C)
A - 0.5
0.2
0.1
0
-0.1
CDS C content
CDS AC
CDS AG
CDS AT
CDS CA
CDS CG
CDS CT
CDS GA
CDS GC
CDS GG
CDS GT
CDS TA
CDS TC
CDS TG
CDS TT
CDS A stat
CDS C stat
CDS G stat
CDS T stat
CDS A runs
CDS C runs
CDS G runs
CDS T runs
3UTR G content
3UTR AC
3UTR AG
3UTR CA
3UTR CT
3UTR GA
3UTR GT
3UTR TC
3UTR TG
3UTR A stat
3UTR G stat
3UTR T stat
3UTR G runs
3UTR T runs
CDS length
ALU density

Figure S3

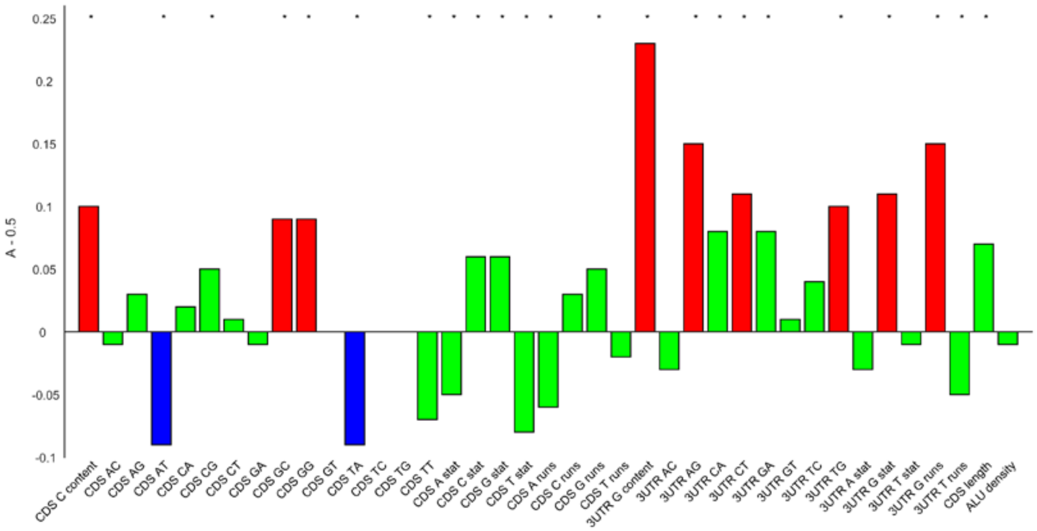

0.25
0.2
0.15
0.1
0.05
0
-0.05
-0.1
A - 0.5
CDS C content
CDS AC
CDS AG
CDS AT
CDS CA
CDS CG
CDS CT
CDS GA
CDS GC
CDS GG
CDS GT
CDS TA
CDS TC
CDS TG
CDS TT
CDS A stat
CDS C stat
CDS G stat
CDS T stat
CDS A runs
CDS C runs
CDS G runs
CDS T runs
3UTR G content
3UTR AC
3UTR AG
3UTR CA
3UTR CT
3UTR GA
3UTR GT
3UTR TC
3UTR TG
3UTR A stat
3UTR G stat
3UTR T stat
3UTR G runs
3UTR T runs
CDS length
ALU density

Figure S4

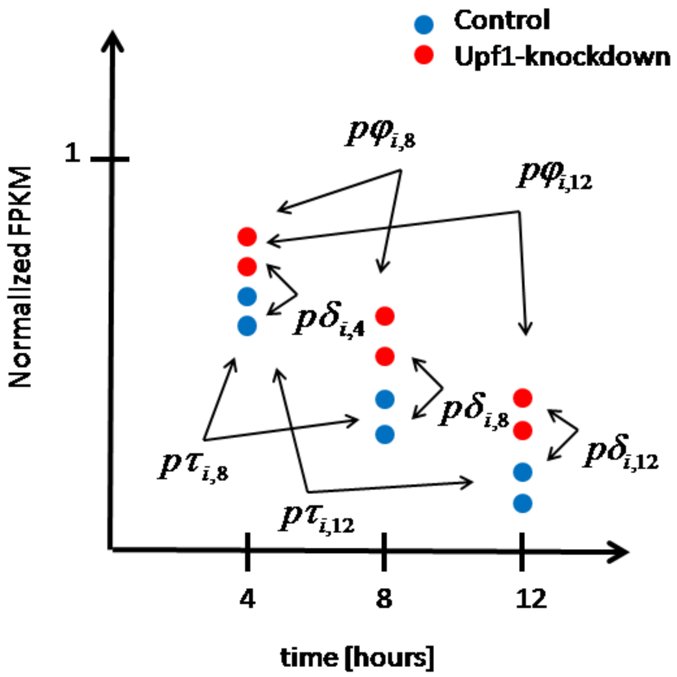

Control
Upf1-knockdown
1
Normalized FPKM
$p\varphi_{i,8}$
$p\varphi_{i,12}$
$p\delta_{i,4}$
$p\delta_{i,8}$
$p\delta_{i,12}$
$p\tau_{i,8}$
$p\tau_{i,12}$
4
8
12
time [hours]
